# Treatment of genitourinary syndrome of menopause with fractional CO₂ laser versus fractional microablative radiofrequency: study protocol for a double-blind, randomised, non-inferiority trial

**DOI:** 10.1186/s13063-026-09865-3

**Published:** 2026-07-03

**Authors:** Cássia Caroline Garcia Dalbem Teles, Catarina Maria Mesquita Garcia Dalbem, Matheus Rodrigues de Souza, Nivaldo Antonio Parizotto

**Affiliations:** 1 Universidade Do Estado de Mato Grosso, Cáceres, Brazil; 2 Universidade Brasil, São Paulo, Brazil

**Keywords:** Genitourinary syndrome of menopause, Fractional CO₂ laser, Fractional microablative radiofrequency, Vaginal atrophy, Non-hormonal therapy, Vaginal Health Index Score, Randomised non-inferiority trial

## Abstract

**Background:**

Genitourinary syndrome of menopause (GSM) affects a significant proportion of postmenopausal women, leading to symptoms such as vaginal dryness, irritation, dyspareunia, and urinary symptoms. Hormonal therapies are not suitable for all patients, particularly those with contraindications to hormone use. This study aims to compare the efficacy and safety of two non-hormonal therapies—fractional CO₂ laser (CO₂ laser) and fractional microablative radiofrequency (MAFRF)—for the treatment of GSM.

**Methods:**

This protocol describes a randomised, double-blind, non-inferiority clinical trial that will enrol 40 postmenopausal women diagnosed with GSM. Participants will be randomised to receive either fractional CO₂ laser or fractional microablative radiofrequency in three monthly sessions. The primary outcome will be the Vaginal Health Index Score (VHIS), assessed 30 days after the last treatment session (day 90). Secondary outcomes include sexual function, GSM symptom severity, and urinary symptoms. Follow-up assessments will be conducted at 120 and 300 days after the last session (days 180 and 365, respectively). Histological analysis of vaginal biopsies will be performed in a predefined subsample of participants as an exploratory analysis.

**Discussion:**

This study is expected to contribute to the comparative understanding of the efficacy and safety of CO₂ laser and MAFRF as non-hormonal therapeutic options for the treatment of GSM, potentially supporting clinical decision-making for women who are not candidates for hormonal therapy.

**Trial registration:**

Brazilian Clinical Trials Registry (ReBEC): RBR-2hgwvgy. The trial was registered retrospectively due to administrative delays in the registration process. However, no outcome data were accessed, analysed, or used to inform any decisions prior to registration, and no interim analyses were performed. Additionally, no modifications were made to the study design, outcomes, or planned analyses after the initiation of data collection. Registered on July 2, 2023.

## Administrative information {1b}

**Table:** 

Item	Description
Primary Registry and Trial Identifying Number {4}	Primary registry: Brazilian Clinical Trials Registry (ReBEC) Trial identification number: RBR-2hgwvgy UTN: U1111-1282-9195 URL: https://ensaiosclinicos.gov.br/rg/RBR-2hgwvgy Date of registration: July 2023 Type of registration: Retrospectively registered trial; no outcome data were analysed prior to registration.
Secondary Identifying Numbers	Not applicable.
Source(s) of Monetary or Material Support	The study did not receive external funding. The devices used in the interventions (fractional CO₂ laser and fractional microablative radiofrequency) belong to the principal investigator. Hospital Regional de Cáceres Dr. Antonio Fontes provided logistical support, clinical infrastructure, and technical staff for the procedures.
Primary Sponsor and contact information {3b}	Primary sponsor: Cássia Caroline Garcia Dalbem Teles Institution: Universidade do Estado de Mato Grosso (UNEMAT) Contact: cassiadalbem@unemat.br
Role of sponsor and funder {3c}	The principal investigator is responsible for the conception of the study, the development of the protocol, and the scientific supervision of study conduct. The principal investigator and the research team remain blinded to group allocation and outcome data during study conduct and during the preparation and submission of the present protocol, without access to statistical results for the purposes of interpretation or methodological decision-making. The institutions involved do not participate in the study design, data collection, data analysis or interpretation, manuscript writing or the decision to submit the manuscript for publication.
Contact for Public Queries	Institutional contact: cassiadalbem@unemat.br Institution: Universidade do Estado de Mato Grosso (UNEMAT)
Contact for Scientific Queries	Principal investigator: Cássia Caroline Garcia Dalbem Teles Institution: Universidade do Estado de Mato Grosso (UNEMAT) Email: cassiadalbem@unemat.br
Public Title	Treatment of genitourinary syndrome of menopause with fractional CO₂ laser versus fractional microablative radiofrequency
Scientific title	Treatment of genitourinary syndrome of menopause with fractional CO₂ laser versus fractional microablative radiofrequency: A double-blind, randomised, non-inferiority clinical trial protocol
Countries of Recruitment	Brazil
Health Condition(s) or Problem(s) Studied	Genitourinary syndrome of menopause
Intervention(s)	Group 1: fractional CO₂ laser will be applied in three monthly sessions. Group 2: fractional microablative radiofrequency will be applied in three monthly sessions.
Key Inclusion and Exclusion Criteria	Inclusion: Postmenopausal women diagnosed with genitourinary syndrome of menopause. Exclusion: Clinical conditions that contraindicate participation in the study or the proposed interventions.
Study Type	Interventional, randomised, double-blind, parallel-group clinical trial with a 1:1 allocation ratio and a non-inferiority design.
Date of First Enrolment	15 February 2023
Sample Size	Forty participants.
Primary outcome(s)	Vaginal Health Index Score (VHIS), will be assessed 30 days after the last treatment session.
Key Secondary outcome(s)	Sexual function (Female Sexual Function Index – FSFI), severity of genitourinary syndrome of menopause symptoms (visual analogue scale – VAS), and urinary symptoms (International Consultation on Incontinence Questionnaire – Short Form – ICIQ-SF) will be assessed at 30, 120, and 300 days after the last treatment session (Days 90, 180 and 365, respectively). The Vaginal Health Index Score (VHIS) will also be assessed at 120 and 300 days, exclusively to evaluate maintenance of the treatment effect over follow-up.
Ethics Review	The study was approved by the Research Ethics Committee of Universidade Brasil. Approval number: 5.357.602.
Individual Trial Participant Data sharing statement	There is no plan to share de-identified individual participant data.

### Protocol version {2}

Version 2.0, January 2026

## Introduction

### Background and rationale {9a}

Genitourinary syndrome of menopause (GSM) is a common condition affecting postmenopausal women and is characterised by vaginal and urinary symptoms, including vaginal dryness, dyspareunia, and urinary discomfort, with a negative impact on quality of life and female sexual function [1]. Although local estrogen therapy is considered the standard treatment, its use is not universal, either due to clinical contraindications, concerns about adverse effects, or poor adherence, which has driven the search for non-hormonal therapeutic alternatives. In addition, observational studies and population-based surveys have shown that a substantial proportion of women with GSM do not initiate or discontinue the use of local oestrogen, even in the absence of formal contraindications, due to safety concerns, personal preferences, or adherence-related difficulties, highlighting a therapeutic gap between first-line treatment options and non-hormonal alternatives [1–3].

Among these alternatives, energy-based therapies have been progressively incorporated into the management of GSM. Fractional CO₂ laser therapy has been associated with improvements in vulvovaginal symptoms and vaginal health indices, with a favourable safety profile [4–6]. Similarly, fractional microablative radiofrequency has demonstrated clinical benefits and good tolerability as a non-hormonal option for the treatment of GSM [7].

Recent clinical evidence, including randomised clinical trials, comparative studies, and histological analyses, indicates that fractional CO₂ laser and fractional microablative radiofrequency produce similar clinical and histological outcomes in the treatment of GSM, without consistent evidence of superiority of one technology over the other. Comparable improvements have been observed in vaginal symptoms, sexual function, vaginal health scores, and histological parameters, with no statistically significant differences between the methods [8–10].

Despite these findings, the available literature still presents methodological limitations, including heterogeneity in study designs and a scarcity of well-designed comparative trials with randomisation, adequate blinding, and structured follow-up. Moreover, there is no consistent evidence of clinical or histological superiority between fractional CO₂ laser and fractional microablative radiofrequency [8–11], which makes the adoption of a non-inferiority design appropriate for comparing interventions that have already demonstrated similar clinical benefits in the treatment of GSM.

From a clinical and public health perspective, issues related to accessibility and technological availability are also relevant when selecting non-hormonal therapies for GSM. Fractional microablative radiofrequency generally allows easier implementation across different healthcare settings when compared with laser-based technologies, which may expand access to treatment, particularly in resource-limited contexts [8, 9].

In this context, the choice of a randomised, double-blind clinical trial with a non-inferiority design was methodologically appropriate to evaluate whether fractional microablative radiofrequency provides clinical efficacy that is not inferior to fractional CO₂ laser, a more extensively studied technology. This design allows assessment of whether a potentially more accessible technology with broader applicability across different healthcare settings can offer similar clinical benefit, without the need to demonstrate superiority, considering the previously observed similarity of clinical and histological outcomes between the two methods [8–11]. The Vaginal Health Index Score (VHIS) was selected as the primary outcome because it integrally reflects clinically relevant changes in vaginal health and has been widely used in previous studies evaluating interventions for GSM [4, 5, 8].

### Explanation for the choice of comparator {9b}

Fractional CO₂ laser was selected as the comparator because it is one of the most extensively studied and widely used non-hormonal energy-based therapies for the treatment of genitourinary syndrome of menopause (GSM), and is frequently regarded as a clinical reference in this context [4–6].

Fractional microablative radiofrequency was chosen as the investigational intervention due to its greater potential for accessibility and incorporation across different healthcare settings. Early evidence from pilot studies has demonstrated relevant clinical benefits and an acceptable safety profile in the treatment of GSM [7], justifying its comparative evaluation against fractional CO₂ laser.

### Objectives {10}

#### Primary objective

To evaluate whether fractional microablative radiofrequency demonstrates clinical efficacy that is non-inferior to fractional CO₂ laser in the treatment of genitourinary syndrome of menopause, using the Vaginal Health Index Score (VHIS) as the primary outcome.

#### Secondary objectives

To evaluate and compare between groups: sexual function, assessed using the Female Sexual Function Index (FSFI); severity of genitourinary symptoms, assessed using a visual analogue scale; urinary symptoms, assessed using the International Consultation on Incontinence Questionnaire – Short Form (ICIQ-SF); maintenance of the clinical effect over follow-up; the occurrence of adverse events and the safety of the interventions, including procedure-related local and systemic effects; and an exploratory histological analysis of vaginal biopsies in a subsample of participants.

## Methods: patient and public involvement and trial design

### Patient and public involvement {11}

Patients and members of the public were not involved in the study design, formulation of the research questions, selection of outcomes, conduct of the study, or data analysis. Participants did not contribute to the development of the protocol or to the writing of the manuscript.

Participants were individually informed about the study objectives, procedures, potential risks and benefits, and their right to withdraw consent at any time, as described in the informed consent section.

### Trial design {12}

This study is a single-centre, interventional, randomised, controlled, double-blind, parallel-group, non-inferiority clinical trial. Participants are randomised to receive three monthly sessions of fractional CO₂ laser or fractional microablative radiofrequency in a 1:1 allocation ratio.

## Methods: participants, interventions and outcomes

### Trial setting {13}

The trial will be conducted at Hospital Regional de Cáceres Dr Antonio Fontes, located in the city of Cáceres, Mato Grosso, Brazil. This is a public tertiary-level hospital affiliated with the Brazilian Unified Health System (SUS), providing specialised outpatient care and serving as a teaching and research centre. 

#### Characteristics of the people who are needed for the trial

**Table:** 

Characteristic	The people we would expect to see included
Age	Women aged **50 to 69 years**, corresponding to the age range with the highest prevalence of genitourinary syndrome of menopause. This range reflects the clinical target population of the study and is consistent with the predefined inclusion criteria
Sex	**Female.** The study includes only individuals of the female sex, as genitourinary syndrome of menopause is a condition specific to women
Gender	**Women**. No other gender identities were anticipated in the protocol, as the condition studied and the eligibility criteria are based on biological characteristics related to female sex
Race, ethnicity and ancestry	**Not restricted and self-reported by participants**. No inclusion or exclusion criteria were applied based on race, ethnicity, or ancestry. The racial and ethnic composition reflects the population served by the local public healthcare system
Socioeconomic status	**Users of the public healthcare system (SUS)**. Participants are recruited from a public tertiary hospital, indicating a population predominantly of low to middle socioeconomic status. No formal socioeconomic stratification criteria were applied
Geographic location	**Brazil**, with recruitment conducted in the city of **Cáceres, Mato Grosso**, at a public tertiary hospital. The population is predominantly urban, with possible inclusion of participants from surrounding rural areas within the hospital’s catchment area
Other characteristics relevant to the trial	Women in **physiological postmenopause**, with a clinical diagnosis of genitourinary syndrome of menopause, without recent use of hormonal therapies or prior vaginal laser or radiofrequency treatments, in accordance with the protocol eligibility criteria

### Eligibility criteria for participants {14a}

#### Inclusion criteria

Women will be eligible if they meet all of the following criteria:

- Physiological postmenopause (amenorrhea > 12 months), with FSH > 40 U/L and estradiol ≤ 25 pg/mL;
- Age between 50 and 69 years;
- Presence of at least one symptom of genitourinary syndrome of menopause, including vaginal dryness, burning, itching, dyspareunia, and/or mild dysuria;
- Negative cervical cytology for neoplasia within the previous 6 months (or according to current screening recommendations for women aged 65 years or older).

#### Exclusion criteria

Women will be excluded if they:

- Decline participation;
- Have used systemic or local hormonal therapy within the past 6 months;
- Have used non-hormonal vaginal lubricants or moisturisers within the past month;
- Have an active genital infection or suspected malignancy;
- Have a history of genital herpes without adherence to antiviral prophylaxis (e.g. acyclovir);
- Have severe comorbidities (autoimmune diseases, HIV, hepatitis B or C, neoplasms, thrombophilias, collagen disorders) or chronic use of corticosteroids or immunosuppressive agents;
- Have stage II or III vaginal prolapse (POP-Q);
- Have a history of photosensitivity disorders;
- Have previously undergone vaginal CO₂ laser or radiofrequency treatment; and
- Have participated in another clinical trial within the past 12 months.

### Eligibility criteria for sites and those delivering interventions {14b}

The trial will be conducted at a single site, Hospital Regional de Cáceres Dr Antonio Fontes, a public tertiary-level hospital affiliated with the Brazilian Unified Health System (SUS), with adequate infrastructure for specialised outpatient care and for performing the procedures specified in the protocol.

The interventions will be performed by a gynaecologist with specific training and prior clinical experience in the use of fractional CO₂ laser and fractional microablative radiofrequency. No other healthcare professional will be involved in the delivery of the interventions.

### Who will take informed consent? {32a}

Informed consent was obtained prior to enrolment and allocation, by means of a written document signed by the participant and the investigator responsible for the study. The consent process took place during an outpatient visit, in a private setting, with a detailed explanation of the study objectives, the procedures involved, the potential risks and benefits, and the participant’s right to withdraw at any time.

Participants were given adequate time for reflection and were allowed to take the informed consent form home for review before returning for eligibility confirmation and potential enrolment in the study. Upon signing, a copy of the consent form was provided to each participant.

A subgroup of participants was randomly selected to undergo vaginal biopsies before and after treatment for histopathological evaluation. This possibility was explained in advance, and specific additional consent for this procedure was obtained prior to allocation. No intervention or study-specific procedure was initiated before formal informed consent was obtained.

### Additional consent provisions for collection and use of participant data and biological specimens {32b}

Participants provided informed consent, through the informed consent form, for the use of their clinical data and biological samples (vaginal biopsies) strictly for the purposes of this study, limited to histopathological analysis using haematoxylin–eosin (HE) and Masson's trichrome staining. The consent included authorisation for temporary storage of the samples until completion of the analysis, including potential reprocessing or quality control prior to statistical evaluation. After this period, all samples will be discarded. No additional use is authorised. Any future use will require new ethics approval and specific participant consent.

## Intervention and comparator

### Intervention and comparator description {15a}

#### Fractional CO₂ laser group (comparator)

Treatment was performed using the MonaLisa Touch device (SmartXide2 V2LR, DEKA, Italy), with a 360° vaginal probe for intravaginal application. Three monthly sessions were conducted, with 30-day intervals between sessions.

The application parameters were 40 watts of power, 1000 µs dwell time, 1000 µm spacing, and using the DekaPulse mode. Ablation depth was adjusted according to the clinical severity of vulvovaginal atrophy, using Stack 2 for severe atrophy, Stack 3 for moderate atrophy, and Stack 4 for mild atrophy.

The vestibular region was treated at each session using a specific vulvar probe, with a power setting of 20 watts, also in DekaPulse mode.

#### Fractional microablative radiofrequency group (intervention)

Treatment was performed using the Wavetronic 6000 Touch device in association with the Megapulse HF FRAXX system (Loktal Medical Electronics, Brazil), with a disposable vaginal probe containing 64 microneedles (200 µm in diameter and 1 mm in length).

Three monthly sessions were performed, with 30-day intervals between sessions. The first session used the Low Energy Soft Treatment mode (40 Hz), and subsequent sessions used the Medium Energy Soft Treatment mode (60 Hz). Energy was delivered in a fractional and randomised pattern across microneedle columns, in order to avoid thermal summation.

The vaginal mucosa was treated in all sessions. The vestibular area was addressed at each application using the low-energy mode (40 Hz).

#### Execution and standardisation of procedures

All procedures were performed by the same interventionist physician, a board-certified gynaecologist with more than 20 years of clinical experience in gynaecology and prior formal training in the use of energy-based technologies for vaginal applications, including fractional CO₂ laser and fractional microablative radiofrequency.

Before study initiation, a pre-study standardisation phase (run-in) was conducted, during which the interventionist performed complete procedures using both technologies. This phase aimed to align technical parameters, application sequence, anatomical approach, and step-by-step execution of the interventions, ensuring consistency, reproducibility, and fidelity to the study protocol.

The physician responsible for delivering the interventions did not participate in clinical outcome assessment or statistical data analysis, contributing to the maintenance of study blinding.

The principal investigator, also a gynaecologist with extensive clinical experience, was responsible for clinical evaluations and outcome assessment. Prior to participant recruitment, she underwent specific training for standardised assessment of climacteric patients and application of the Vaginal Health Index Score (VHIS). This training included repeated assessments in climacteric patients at two distinct time points (baseline and approximately 30 days), aiming to align instrument application and minimise intra-observer variability, without constituting a formal reproducibility study.

### Criteria for discontinuing or modifying allocated intervention/comparator {15b}

The allocated intervention may be discontinued or modified for an individual trial participant under the following circumstances:

- Explicit request by the participant to discontinue treatment at any time and for any reason;
- Occurrence of a clinically relevant adverse event related to the intervention, including but not limited to severe pain, abnormal bleeding, local infection, or tissue injury;
- Development of an intercurrent clinical condition not related to the intervention—such as systemic infection, hospital admission, acute clinical condition, or need for urgent surgical procedure—that, in the physician’s judgement, contraindicates continued participation in the study;
- Non-compliance with protocol criteria after initiation of the intervention, including the use of systemic or local hormone therapy during follow-up;
- Recommendation by the independent safety monitor based on safety assessment.

In accordance with the double-blind design of the study, the assessment of adverse events and initial safety-related decisions may involve direct communication between the physician member of the independent safety monitor and the physician responsible for delivering the interventions, without unblinding the principal investigator or the outcome assessment team.

The principal investigator will be formally informed only in cases where the recommendation requires discontinuation of the intervention, protocol modification, or trial interruption, in accordance with ethical and regulatory requirements.

### Strategies to improve adherence to intervention/comparator {15c}

Strategies were implemented to promote adherence to the allocated interventions and follow-up visits. Participants were contacted by telephone calls or electronic messaging (e.g. WhatsApp), according to individual preference, to remind them of scheduled treatment sessions and follow-up assessments.

Communication was personalised and delivered in clear, supportive language, emphasising the importance of completing all treatment sessions and study visits. The research team remained available throughout the follow-up period to address questions, provide guidance, and offer additional support when needed.

Attendance at each intervention session was systematically recorded in a secure electronic database. In cases of missed appointments, participants were promptly contacted to identify barriers and to reschedule the session whenever possible. This active monitoring allowed early identification of adherence difficulties and facilitated individualised strategies to support continued participation.

### Concomitant care permitted or prohibited during the trial {15d}

During the study period, the use of any hormonal therapy, either systemic or topical, including vaginal oestrogens, hormonal modulators, or phytoestrogen-containing products, will not be permitted. The use of vaginal lubricants, moisturisers, creams, or other non-hormonal topical products that could interfere with the effects of the evaluated interventions will also be prohibited.

The use of systemic medications, such as antibiotics, analgesics, or anti-inflammatory drugs, will be permitted when clinically indicated, provided that they do not have a direct impact on the primary or secondary study outcomes. All medications used during follow-up will be recorded and reviewed by the clinical team.

If the need for therapies with potential interference in the study outcomes is identified—such as hormonal therapy or vaginal topical products—the participant’s continuation in the protocol will be reassessed. When significant interference is confirmed, the participant may be withdrawn from the study according to criteria predefined in the protocol.

### Ancillary and post-trial care {34}

During the conduct of the study, participants will have access to ancillary clinical care whenever necessary, particularly for the assessment and management of adverse events or clinical intercurrences potentially related to the interventions. Such care will be provided at the study site and will be offered at no cost to the participants.

Participants will be followed according to the study protocol for a total period of up to 12 months after treatment initiation (first treatment session), including scheduled clinical assessments to monitor therapeutic response and to identify any late adverse events. This follow-up period is an integral part of the study design and does not include additional therapeutic interventions after completion of the treatment sessions.

In the event that any harm or adverse event related to study participation is identified, appropriate medical care will be provided to the participant without financial burden, in accordance with institutional policies and applicable ethical guidelines.

No financial compensation is planned for participation in the study. After completion of the protocol-defined follow-up period, no additional study-specific follow-up will be conducted, and participants will be advised to continue routine clinical care within the usual healthcare system.

### Outcomes {16}

#### Primary outcome

The primary outcome will be vaginal health, measured using the Vaginal Health Index Score (VHIS).

- Measurement variable: total VHIS score
- Instrument description: the VHIS consists of five clinical domains—vaginal mucosal elasticity, vaginal pH, vaginal fluid volume, epithelial integrity, and vaginal moisture. Each domain is assessed by a trained examiner and scored on an ordinal scale from 1 to 5, in which 1 represents the most unfavourable condition and 5 represents the most favourable condition of the respective clinical parameter. The total score results from the simple sum of the five domains, ranging from 5 to 25 points, with higher scores indicating better vaginal health. Changes in the total VHIS score are clinically interpreted as variations in the severity of vaginal atrophy and related functional parameters, reflecting perceptible changes in vaginal health.
- Analysis metric: change in score from baseline (ΔVHIS).
- Aggregation method: mean and standard deviation or median and interquartile range, according to data distribution.
- Primary time point: 30 days after the last treatment session (day 90), defined as the main time point for primary outcome analysis.

Additional VHIS assessments will be performed at later time points exclusively to evaluate maintenance of effect over follow-up and will not be considered for the primary outcome.

#### Secondary outcomes

Secondary outcomes include:

1. Female sexual function assessed using the Female Sexual Function Index (FSFI).
2. Measurement variables: total FSFI score and domain scores
3. Analysis metric: change from baseline
4. Method of aggregation: mean and standard deviation (or median and interquartile range)
5. Severity of genitourinary symptoms, including vaginal dryness, burning, itching, and dyspareunia, assessed using a visual analogue scale (VAS).
6. Measurement variable: score in centimetres (0–10)
7. Analysis metric: change from baseline
8. Method of aggregation: mean and standard deviation
9. Urinary symptoms assessed using the International Consultation on Incontinence Questionnaire – Short Form (ICIQ-SF).
10. Measurement variable: total questionnaire score
11. Analysis metric: change from baseline
12. Method of aggregation: mean and standard deviation
13. Maintenance of clinical effect, evaluated through the longitudinal evolution of VHIS, FSFI, VAS, and ICIQ-SF scores over follow-up.
14. Safety of interventions, assessed by the occurrence of local or systemic adverse events related to the procedures, recorded descriptively throughout the study.
15. Vaginal histological assessment, performed as an exploratory analysis in a predefined subsample of participants. Given the small sample size, these findings will be interpreted with caution and considered hypothesis-generating, not allowing for definitive inferences.
16. Measurement variables: histopathological changes in vaginal epithelium and connective tissue
17. Method: analysis of vaginal biopsies stained with haematoxylin–eosin (HE) and Masson’s trichrome
18. Objective: exploratory evaluation of tissue response to the interventions

#### Time points of assessment

All outcomes will be assessed at the following time points:

- Baseline (before the first treatment session)
- 30 days after the last treatment session (day 90)
- 120 days after the last treatment session (day 180)
- 300 days after the last treatment session (day 365)

The time point at 30 days after the last treatment session is defined as the primary time point for analysis of the primary outcome. The remaining time points are intended for evaluation of maintenance of effect and longitudinal analysis.

#### Statistical analysis

Statistical analysis of outcomes had not been performed at the time of submission of this protocol.

### Harms {17}

Adverse events (AEs) are defined as any unfavourable medical signs, symptoms, or conditions occurring during the study period, regardless of their causal relationship with the interventions. Serious adverse events (SAEs) are defined as events resulting in death, life-threatening conditions, hospitalisation, persistent or significant disability, or requiring immediate medical intervention.

Based on previous studies and the nature of the interventions, anticipated adverse events mainly include mild and transient local effects, such as vaginal burning, discomfort, vaginal discharge, local hyperaemia, mild bleeding, and pain during or after the procedure.

All adverse events will be systematically assessed from the time of informed consent until completion of follow-up. As part of an active monitoring strategy, participants will be contacted by the study team approximately 5 days after each intervention session to facilitate early identification of symptoms or intercurrent events.

For each adverse event, the following information will be recorded: type of event, severity, duration, need for medical treatment, and potential causal relationship with the intervention.

Causality assessment will be performed by an independent physician acting as an independent safety monitor, based on review of clinical records and participant-reported information. No standardised adverse event coding system (such as MedDRA) will be applied; events will be classified descriptively according to clinical judgement.

All recorded adverse events will be reported in publications arising from this study. Any serious adverse event, if identified, will be promptly reported to the Research Ethics Committee in accordance with applicable regulatory requirements.

### Participant timeline {18}

Participants followed a structured schedule of visits throughout the study, comprising a pre-screening phase, formal inclusion, the intervention period, and post-intervention follow-up.

#### Pre-screening phase (initial clinical visit)

During routine outpatient gynaecological care, potentially eligible women were identified by the attending physician. At this stage, the study was briefly introduced, including its objectives, procedures, and potential risks and benefits. Participants were provided with the informed consent form (ICF) for home review, and laboratory and clinical examinations required to confirm eligibility were requested. No study data were collected and no study-related procedures or interventions were performed during this phase.

#### T0—inclusion visit (return with examinations)

At the follow-up outpatient visit, designated as T0, all inclusion and exclusion criteria were assessed based on the submitted examinations and clinical evaluation. The informed consent form was reviewed again, any remaining questions were clarified, and, after formal agreement, the consent was signed. From this point onward, the participant was officially enrolled in the study and allocated to one of the intervention groups according to the previously generated randomisation sequence.

No questionnaires were administered, no outcome assessments were performed, and no intervention was delivered during this visit.

#### Day 1—clinical baseline assessment and first intervention

On day 1, immediately before the first treatment session, continued consent was confirmed. Baseline outcome assessments were then performed, including administration of the Female Sexual Function Index (FSFI), the visual analogue scale (VAS) for genitourinary symptoms, the International Consultation on Incontinence Questionnaire – Short Form (ICIQ-SF), and clinical evaluation using the Vaginal Health Index Score (VHIS). Only after completion of all baseline assessments was the first session of the allocated intervention (fractional CO₂ laser or fractional microablative radiofrequency) performed. In a predefined subgroup of participants, a pre-intervention vaginal biopsy was also obtained during this visit.

#### Interventions

Participants received three monthly sessions of the allocated intervention:

Day 1: first session

Day 30: second session

Day 60: third session

Clinical assessments and questionnaires were reapplied immediately before each intervention session, allowing longitudinal monitoring of treatment response during the intervention period.

#### Post-intervention follow-up

After completion of the three intervention sessions, participants were followed up at visits defined in relation to the last treatment session:

- 30 days after the last session (day 90)
- 120 days after the last session (day 180)
- 300 days after the last session (day 365)

At each follow-up visit, the VHIS, FSFI, VAS, and ICIQ-SF were reassessed to evaluate maintenance of the clinical effect and to identify any late adverse events. In a predefined subsample, a vaginal biopsy was performed at 120 days after the last session (day 180) as an exploratory histological analysis, with findings interpreted as hypothesis-generating due to the limited sample size.

**Fig. 1:**
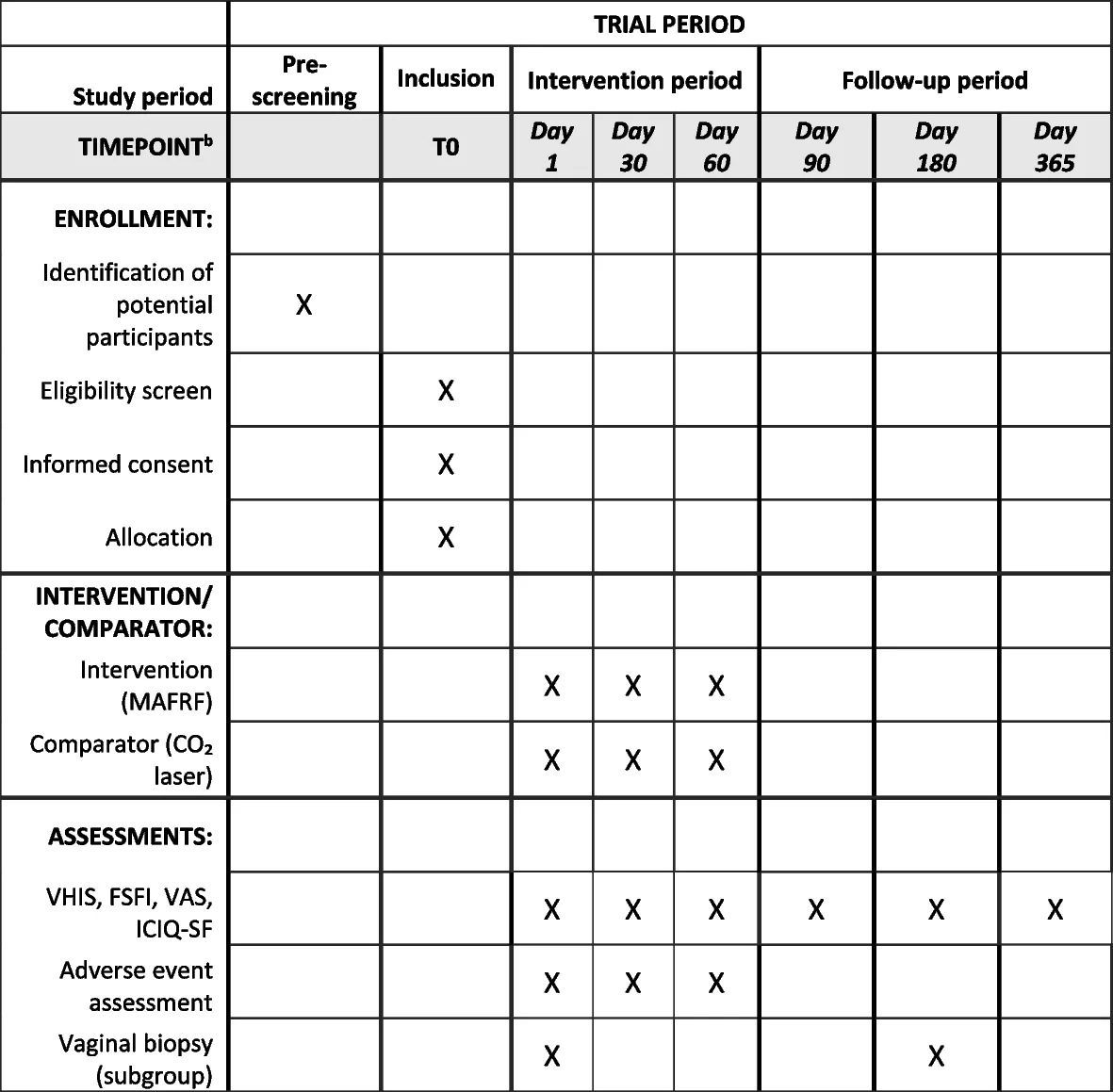
Schedule of enrolment, interventions, and assessments (SPIRIT 2025) [12]. Abbreviations: MAFRF, microablative fractional radiofrequency; CO₂ laser, carbon dioxide laser; VHIS, Vaginal Health Index Score; FSFI, Female Sexual Function Index; VAS, visual analogue scale; ICIQ-SF, International Consultation on Incontinence Questionnaire–Short Form. Informed consent: obtained at T0 and confirmed prior to the first intervention. Adverse events: actively assessed 5 days after each intervention session. Clinical assessments: include VHIS, FSFI, VAS, and ICIQ-SF. Vaginal biopsy: performed in a predefined subsample before the first intervention (day 1) and during follow-up (day 180)

The schedule of enrolment, interventions, and assessments is presented in Fig. 1, in accordance with the SPIRIT 2025 recommendations.

### Sample size {19}

The sample size calculation was based on a non-inferiority randomised clinical trial design, with the Vaginal Health Index Score (VHIS) as the primary outcome. The following statistical assumptions were adopted: a one-sided type I error (α) of 0.025; a type II error (β) of 0.10, corresponding to 90% statistical power; a non-inferiority margin of 3 points on the VHIS; an estimated population standard deviation of 2.5 points; and an expected mean difference of 0 between groups. The calculation followed the method described by Flight and Julious [13], with parameters entered into the SampSize® application (https://app.sampsize.org.uk).

The non-inferiority margin of 3 points on the Vaginal Health Index Score (VHIS) was defined based on clinical considerations and on the magnitude of effect observed in previous studies. The VHIS is a composite scale ranging from 5 to 25 points, derived from the sum of five clinically relevant parameters of vaginal health (elasticity, vaginal fluid volume, pH, epithelial integrity, and hydration), with scores equal to or greater than 15 generally considered compatible with a non-atrophic vaginal condition [4, 5]. An absolute difference of 3 points in the VHIS can be interpreted as a clinically perceptible change across one or more domains, reflecting, for example, an improvement from moderate to mild atrophy or the normalisation of specific vaginal parameters, such as hydration or epithelial integrity.

Although the VHIS does not have a formally validated minimal clinically important difference, it is a widely used outcome in clinical trials and prospective studies evaluating therapies for genitourinary syndrome of menopause, in which treatment effects are commonly interpreted based on absolute changes in the score. Clinical trials and systematic reviews assessing effective therapies, such as fractional CO₂ laser and other energy-based interventions, have reported mean improvements in the VHIS that are substantially greater, frequently in the range of 5 to 10 points compared with baseline [4, 5, 14].

In this context, the choice of a non-inferiority margin of 3 points preserves a clinically relevant proportion of the expected treatment effect, consistent with recommendations for non-inferiority margin justification, representing a small variation in the overall score. Accordingly, this margin constitutes a conservative and clinically plausible threshold for the assessment of non-inferiority, ensuring the maintenance of a clinically meaningful benefit.

Based on these assumptions, a minimum of 32 analysable participants (16 per group) was estimated.

The sample size calculation considered as the primary scenario the requirement of these 32 analysable participants, without incorporating attrition into the initial calculation. To accommodate potential losses to follow-up, a total recruitment target of 40 participants was planned, corresponding to an anticipated dropout rate of 20% (40 planned vs. 32 analysable participants).

In addition, a sensitivity assessment of statistical power for the non-inferiority hypothesis was conducted to evaluate the robustness of the sample size under plausible attrition scenarios, while keeping all original assumptions unchanged. In this assessment, the estimated power was 0.91 with 16 participants per group (*N* = 32), 0.86 with 14 participants per group (*N* = 28; approximately 12.5% loss), and 0.84 with 13 participants per group (*N* = 26; approximately 18.8% loss), indicating that the study retains adequate statistical power even under moderate levels of loss to follow-up (dropout) (Table 1).

**Table 1:** Sensitivity analysis of statistical power under attrition scenarios

Participants per group	Total *N*	Estimated loss (%)	Power
16	32	0%	0.91
14	28	12.5%	0.86
13	26	18.8%	0.84

Nevertheless, considering the observed loss to follow-up, it is acknowledged that the final number of participants with primary outcome assessment may be lower than initially planned, which may reduce the statistical power to demonstrate non-inferiority. Therefore, the findings will be interpreted with caution, taking this limitation into account.

Sensitivity analyses will be used to assess the robustness of the findings under different assumptions regarding missing data.

### Recruitment {20}

Participant recruitment was planned through consecutive screening of women attending the gynaecology outpatient clinic at Hospital Regional de Cáceres Dr Antonio Fontes. Potentially eligible participants were approached in person during previously scheduled gynaecological consultations, at which time they received detailed information about the study objectives, proposed procedures, and potential risks and benefits.

As a strategy to promote adherence and achieve the planned sample size, eligible women were given the opportunity to take the informed consent form home for further consideration, allowing sufficient time for reflection and clarification of any questions with the research team before making a final decision regarding participation. The recruitment team was trained to provide clear and standardised information in order to minimise concerns related to the study procedures.

Participant recruitment took place after approval by the Research Ethics Committee. The screening phase of potential participants occurred between October 2022 and 13 February 2023. Formal inclusion of the first participant in the study occurred on 15 February 2023, following confirmation of eligibility criteria and signing of the informed consent form. Recruitment was concluded after reaching the predefined sample size, which was considered sufficient to achieve the statistical power specified in the protocol.

## Assignment of interventions: randomisation

### Sequence generation: who will generate the sequence {21a}

The random allocation sequence was generated electronically by an independent member of the research team who was not involved in data collection, outcome assessment, or clinical interventions. Randomisation was performed using the Sealed Envelope™ platform (https://www.sealedenvelope.com), with a 1:1 allocation ratio between the two study groups.

### Sequence generation: type of randomisation {21b}

Simple randomisation was used, with a 1:1 allocation ratio between the two study groups. No stratification factors or restricted randomisation methods were applied.

### Allocation concealment mechanism {22}

Allocation concealment was ensured through electronic randomisation with restricted access, under the responsibility of a member of the research team not involved in data collection, outcome assessment, or clinical interventions. The allocation sequence was operationalised using sequentially numbered, sealed, opaque envelopes.

The envelopes were accessed only by the physician responsible for the intervention and the intervention nurse, immediately prior to the intervention, after completion of baseline assessment. This procedure ensured allocation concealment and preserved the integrity of the double-blind study design.

### Implementation {23}

Participant enrolment was performed by the principal investigator, who conducted recruitment, confirmed eligibility criteria, and obtained informed consent, without access to the random allocation sequence.

Assignment of participants to the intervention groups was performed after enrolment (T0), based on the previously generated random allocation sequence. Access to the allocation was restricted exclusively to the physician responsible for delivering the intervention and the intervention nurse, only immediately prior to the intervention and solely for operational purposes.

No personnel involved in participant enrolment, data collection, outcome assessment, or statistical analysis had prior or unrestricted access to the random allocation sequence.

## Assignment of interventions: blinding

### Who will be blinded {24a}

This study was designed as a randomised, controlled, double-blind clinical trial. Trial participants, the investigator responsible for clinical assessments and outcome collection, the pathologist who evaluated the biopsy specimens, the physician responsible for safety monitoring, and all other members of the clinical and care teams involved in participant follow-up remained blinded to the assigned intervention.

Only the physician responsible for delivering the intervention and the interventional nurse were aware of treatment allocation, solely due to technical requirements for performing the procedures; neither was involved in data collection, outcome assessment, or statistical analysis.

### How will be blinding be achieved {24b}

Blinding was achieved through standardised procedures designed to minimise perceptible differences between interventions and to prevent identification of the assigned treatment. All sessions were conducted in a dedicated procedure room equipped with both therapeutic devices (fractional CO₂ laser and fractional microablative radiofrequency), regardless of group allocation.

During the procedures, participants wore noise-cancelling headphones and were positioned so as not to visualise the device in use, through the use of visual barriers and surgical draping. These measures were intended to reduce auditory and visual cues that could potentially distinguish between interventions.

Blinding procedures were explained in advance during the informed consent process. To date, no participants have reported adverse events related to the use of noise-cancelling headphones or visual barriers during the procedure. Participants were informed that they could decline or request adjustments to the blinding methods at any time, without any impact on their participation in the study or on the clinical care provided.

The team responsible for delivering the interventions had no involvement in clinical assessments or outcome evaluation and did not communicate with outcome assessors, further supporting the maintenance of blinding throughout the study.

It is acknowledged that, due to the distinct characteristics of the energy-based interventions, participants may perceive differences during the procedures, which may partially limit the effectiveness of blinding.

Outcome assessments were conducted using standardised instructions and validated instruments, with trained evaluators to minimise measurement bias, particularly for subjective outcomes. No formal assessment of participants’ perception of treatment allocation was planned in the study protocol.

### Procedure for unblinding if needed {24c}

Unblinding is not planned as part of routine study procedures, and the trial is designed to maintain blinding of investigators and outcome assessors throughout follow-up whenever possible. In the event of an adverse event, clinical management will be led by the physician responsible for delivering the intervention, who is already aware of the assigned treatment, in consultation with the physician responsible for safety monitoring.

Formal unblinding of additional study personnel is not routinely anticipated and will be undertaken only if strictly required to ensure participant safety, such as in the case of a serious adverse event or an unexpected clinical situation requiring management dependent on the type of intervention delivered. In such exceptional circumstances, unblinding will be restricted to the affected participant only and disclosed solely to the minimum necessary personnel through controlled access to the randomisation record. All unblinding events will be fully documented, including the reason, date, and personnel involved, and reported according to the study safety monitoring procedures and applicable ethical requirements.

## Data collection and management

### Plans for assessment and collection of outcomes {25a}

The primary outcome was assessed using the Vaginal Health Index Score (VHIS), which comprises five clinical parameters: vaginal mucosal elasticity, vaginal pH, vaginal fluid volume, epithelial integrity, and vaginal moisture. The total VHIS score was used for comparative analysis between the groups. Clinical assessment was performed by the physician researcher, who was previously trained and blinded to group allocation.

Secondary outcomes were assessed as follows: sexual function was evaluated using the Female Sexual Function Index (FSFI), a validated self-administered questionnaire covering the domains of desire, arousal, lubrication, orgasm, satisfaction, and pain; genitourinary symptoms (vaginal dryness, burning, itching, and dyspareunia) were assessed using a 10-cm visual analogue scale (VAS); self-reported sexual satisfaction was also assessed using a VAS, in which 0 represents no satisfaction and 10 represents maximum satisfaction; and urinary symptoms were evaluated using the International Consultation on Incontinence Questionnaire – Urinary Incontinence Short Form (ICIQ-SF), a validated instrument for the assessment of urinary incontinence.

Patient-reported outcome questionnaires (FSFI, VAS, and ICIQ-SF) were completed by the participants themselves, following standardised prior instructions provided in advance by the assisting healthcare team present in the room. The assisting team remained available during questionnaire completion to clarify any questions, without interfering with participants’ responses. All professionals involved in instructing participants underwent prior training, with standardised guidance procedures to ensure uniform application of the instruments and consistency in data collection.

Histopathological evaluation was performed in a subgroup of participants (*n* = 4 per group) using vaginal biopsies stained with haematoxylin–eosin (HE) and Masson’s trichrome, allowing assessment of epithelial changes and tissue response. The slides were analysed by an experienced pathologist who was blinded to the allocated intervention.

All assessments were conducted at predefined time points: at baseline, immediately before the first intervention session (day 1); 30 days after each intervention session (days 30, 60, and 90); and at late follow-up assessments on days 180 and 365, corresponding to approximately 120 and 300 days after completion of the last intervention session, respectively.

Only validated instruments were used for the assessment of clinical and patient-reported outcomes. Prior to study initiation, standardised training of all involved professionals was conducted based on a predefined operational protocol, covering criteria for application, data recording, and quality control. Data were recorded using standardised case report forms specifically developed for this study, which are available upon reasonable request.

### Plans to promote participant retention and complete follow-up {25b}

Adherence to the study protocol and participant retention were promoted through close follow-up, with clearly defined roles among the teams involved, under the supervision of the principal investigator, who was responsible for the clinical assessments and overall study follow-up.

A blinded administrative support team, with no access to group allocation, was responsible for scheduling intervention sessions and follow-up visits. This team used telephone reminders and electronic messaging (e.g. WhatsApp) and offered flexible scheduling according to participants’ availability. The administrative team had no access to information regarding the intervention delivered.

For the management of intercurrent events and adverse events, participant contact was conducted by the intervention nurse, who was not blinded to allocation. The intervention nurse was responsible for guiding participants, administering questionnaires related to adverse events, and assessing the nature of reported complaints. Depending on the type of concern, contact was forwarded to the appropriate professional, either the principal investigator (for clinical or follow-up-related issues) or the intervention physician (for procedure-related issues), without disclosure of intervention-related information to the principal investigator, when not relevant. This communication workflow was implemented as an operational strategy to ensure maintenance of blinding of the investigator responsible for clinical assessments and outcome evaluation.

As an active monitoring and participant engagement strategy, a standardised contact was planned 5 days after each procedure, via electronic messaging, to assess participant well-being and to investigate the occurrence of potential adverse events.

In cases of technical difficulties during the intervention, management was determined by the intervention physician, who documented the event in a standardised manner, without contact with the principal investigator, in order to preserve blinding. All adverse events were recorded using a specific form. In the event that serious adverse events occurred, it was planned that causality assessment would be conducted by an independent physician acting as an independent safety monitor, and that such events would be reported immediately to the Research Ethics Committee, in accordance with regulatory requirements.

It was planned that, even in cases of total or partial interruption of the intervention, or protocol deviations, participants would be encouraged to continue clinical follow-up whenever possible. In these situations, primary and secondary outcome data (Vaginal Health Index Score; Female Sexual Function Index; visual analogue scale for genitourinary symptoms and sexual satisfaction; and International Consultation on Incontinence Questionnaire – Urinary Incontinence Short Form) would continue to be collected at the predefined time points, in accordance with the intention-to-treat principle.

### Data management {26}

Clinical and outcome data collection and data entry were performed using standardised electronic forms, implemented on the Google Forms platform and accessed on electronic devices at the time of data collection. Validated questionnaires (FSFI, ICIQ–SF and VAS) were completed directly by participants, with on-site support from a trained member of the assisting healthcare team, limited to technical guidance on form completion and without interference with participants’ responses. Clinical assessments (VHIS) were conducted exclusively by the principal investigator, through gynaecological examination using a vaginal speculum.

Electronic forms were configured to prevent submission with mandatory fields left blank, ensuring data completeness at the time of entry. Standardisation of data entry procedures was reinforced through prior training of all involved personnel, in accordance with the study timeline.

Data were identified using numeric codes, without direct personal identifiers, ensuring participant confidentiality. The electronic database was stored in a secure digital environment, with restricted access throughout the data collection phase, thereby preserving study blinding. The principal investigator remains blinded to group allocation and has not accessed intervention-segregated data to date, maintaining the study under double-blind conditions.

As a data quality control and safety monitoring measure, the independent physician acting as an independent safety monitor had access to the electronic forms for periodic data checks and adverse event monitoring, without sharing this information with the principal investigator.

Full access to the final database is conditional upon submission of this protocol for publication, at which point formal database unlocking is planned to allow statistical analyses. Vaginal biopsy specimens were analysed by a pathologist blinded to allocation, using standard staining techniques (haematoxylin–eosin and Masson’s trichrome).

Data collection and storage using electronic forms were conducted in accordance with institutional standards for data security and data protection. Access to the data was restricted to authorised members of the research team, including the independent safety monitor, through controlled permissions, ensuring confidentiality and integrity of the collected information.

### Confidentiality {33}

Personal information related to study participants was collected and handled with strict confidentiality, in accordance with ethical principles governing research involving human subjects and applicable regulations. At the time of study inclusion, each participant was identified exclusively by a numeric code, and no nominal data or other direct personal identifiers were recorded in the data collection forms or in the databases used for analysis.

Before and during study conduct, operational data required for participant contact (such as contact lists and pre-intervention clinical records) were stored separately from research data, in a secure digital environment, with access restricted to team members responsible for these activities. Outcome data and other research-related information were stored in a secure electronic system (Google Forms), without any possibility of direct participant identification.

During the study, access to research data was restricted and occurred exclusively for safety monitoring purposes by the independent physician acting as an independent safety monitor, without access to participant identities. The principal investigator remains blinded to group allocation and has not accessed intervention-segregated data, thereby maintaining the study under double-blind conditions until submission of this protocol for publication.

Participants’ individual information will be used exclusively for scientific purposes, and no participant will be identifiable in any presentations, reports, or publications arising from the study. After completion of the trial, research data will be stored in a secure location for a minimum period of 5 years, in accordance with regulatory requirements, and will subsequently be securely destroyed.

## Statistical methods

### Statistical methods for primary and secondary outcomes {27a}

Statistical analyses will be conducted according to the intention-to-treat (ITT) principle, including all randomised participants, regardless of protocol adherence or losses during follow-up.

For the primary outcome, vaginal health assessed by the Vaginal Health Index Score (VHIS) 30 days after the last treatment session (day 90), the comparison between groups will be performed by estimating the mean difference (MAFRF minus CO₂ laser), with calculation of a one-sided 97.5% confidence interval. Non-inferiority will be established if the lower bound of the confidence interval remains above the pre-specified non-inferiority margin of − 3 points on the VHIS. A one-sided type I error rate of *α* = 0.025 will be adopted, in accordance with the non-inferiority trial design.

Missing data for the primary and secondary outcomes will be handled using multiple imputation under the assumption of missing at random (MAR). Imputation will be performed using appropriate models for continuous and categorical variables in the R software, incorporating baseline clinical characteristics and available longitudinal data. For participants missing a given assessment, all data corresponding to that time point will be imputed. Final statistical analyses will be conducted using IBM SPSS®, version 27.

Given the non-inferiority design, it is acknowledged that methods for handling missing data, such as multiple imputation, may reduce differences between groups and potentially bias results toward non-inferiority. Therefore, the findings will be interpreted with caution, particularly if the proportion of missing data exceeds the levels initially anticipated.

As a sensitivity analysis, a complete-case analysis will be performed for the primary outcome, including only participants who attended the assessment 30 days after the last treatment session (day 90), regardless of subsequent losses at later follow-up time points (days 180 and 365). This analysis will be conducted without data imputation and will assess the robustness of the non-inferiority conclusion obtained in the primary intention-to-treat analysis.

Additional sensitivity analyses will be conducted to evaluate the robustness of the findings under different assumptions regarding missing data, including complete-case analysis at the primary time point and conservative scenarios for missing outcome handling.

For secondary outcomes, categorical variables will be analysed using Pearson’s chi-squared test or Fisher’s exact test, as appropriate. Continuous variables will be compared using Student’s *t* test (with adjustment for equal or unequal variances) or the Mann–Whitney test when normality assumptions are violated. Longitudinal comparisons over time will be performed using repeated-measures analysis of variance (ANOVA) or the Friedman test when parametric assumptions are not met. For secondary outcomes, a two-sided significance level of *p* < 0.05 will be adopted, with 95% confidence intervals.

Adverse events will be analysed descriptively, with between-group comparisons performed when appropriate.

### Who will be included in each analysis {27b}

All statistical analyses will include all randomised participants, in accordance with the intention-to-treat (ITT) principle. Participants will be analysed in the group originally assigned by randomisation, regardless of protocol adherence, total or partial interruption of the intervention, or occasional missed assessments. This approach will be applied to both the primary and secondary outcomes, ensuring consistency in comparisons between groups.

### How missing data will be handled in the analysis {27c}

Protocol deviations, intervention interruptions, and occasional missed assessments will not result in changes to group allocation, and the original randomisation assignment will be maintained according to the ITT principle.

All missing data for primary and secondary outcomes will be handled using multiple imputation, under the assumption that data are missing at random (MAR), incorporating information available from other follow-up time points and relevant clinical variables.

For the primary outcome—VHIS assessed 30 days post-treatment (day 90)—an additional sensitivity analysis is planned, restricted to participants with complete data at this time point (complete-case analysis), maintaining the original allocation and comparing results with the primary ITT analysis to assess the robustness of the non-inferiority conclusion.

### Methods for additional analyses (e.g. subgroup analyses) {27d}

No formal additional analyses, such as subgroup or adjusted analyses, are planned. Any exploratory analyses that may be conducted will be clearly identified as post hoc and interpreted descriptively, without confirmatory inference.

### Interim analyses {28b}

No formal interim statistical analyses are planned for this trial. Participant safety will be monitored by an independent physician acting as an independent safety monitor, with a focus on identifying serious adverse events or unforeseen risks. No formal statistical stopping rules are defined. If relevant safety concerns are identified, the independent safety monitor may recommend protocol modifications or study termination based on clinical and ethical judgement. 

### Protocol and statistical analysis plan {5}

The study protocol will be made publicly available through publication of this manuscript in Trials. The statistical analysis plan is described in detail within the statistical methods section of this protocol, and no separate standalone statistical analysis plan document is provided.

## Oversight and monitoring

### Composition of the coordinating centre and trial steering committee {3d}

The study is coordinated by a central coordinating centre responsible for the overall organisation, conduct, and supervision of the clinical trial, comprising the principal investigator and a multidisciplinary team trained in clinical research. This team is responsible for participant recruitment, delivery of the interventions, performance of clinical assessments, data collection, and follow-up, ensuring adherence to the protocol and the methodological integrity of the study.

Day-to-day operational conduct of the trial is carried out by a Study Management Group, responsible for monitoring daily activities, logistical control, oversight of protocol adherence, verification of data completeness, and resolution of operational issues. This group meets monthly during the recruitment and follow-up phases.

Scientific and strategic oversight of the study is provided by a Trial Steering Committee, responsible for evaluating trial progress, protocol compliance, recruitment performance, and the overall conduct of the study. The Trial Steering Committee meets at predefined intervals throughout the study or on an ad hoc basis, as required.

Participant safety is monitored by an independent physician acting as an independent safety monitor, responsible for reviewing reported adverse events and issuing recommendations regarding trial continuation. Communication pathways between the clinical team and the independent safety monitor follow predefined procedures to preserve blinding and ensure participant safety.

No outcome adjudication committee was established, as the primary and secondary outcomes were predefined and are based on validated clinical, histological, and patient-reported instruments applied in a standardised manner.

### Composition of the data monitoring committee, its role and reporting structure {28a}

Safety monitoring of the study was conducted by an independent physician with no affiliation to the research team or to any source of study funding and who was blinded to group allocation.

The independent safety monitor was responsible for overseeing participant safety and data integrity, including verification of completeness, consistency, and adequacy of completion of the collected clinical and patient-reported outcomes. The monitor had access to the collected clinical and patient-reported outcome data throughout the study solely for the purposes of safety monitoring and data quality assessment. No interim analyses of efficacy were performed.

The independent safety monitor conducted scheduled monthly reviews of the collected data and also operated in a reactive manner, being additionally consulted in response to the occurrence of relevant adverse events or situations requiring specific evaluation.

Considering the double-blind design of the study, the principal investigator and the team responsible for the assessment of clinical outcomes (VHIS, FSFI, VAS, and ICIQ-SF) remained blinded to group allocation throughout the conduct of the trial. The interventions were delivered by a physician and a nurse who were not blinded, due to technical requirements, both of whom were committed to maintaining blinding of the outcome assessment team.

During the study period, the database containing the collected outcome data remained under the exclusive responsibility of the independent safety monitor and was not accessible to the principal investigator or the outcome assessors. Release of the database for analysis occurred only after completion of participant follow-up and after submission of the study protocol for publication, thereby preserving blinding and methodological integrity.

In the event of adverse events, direct communication was established between the independent safety monitor and the physician responsible for delivering the interventions, with the aim of evaluating clinical management while preserving blinding of the principal investigator. The principal investigator was informed only in situations requiring interruption or modification of the study.

The independent safety monitor did not have authority to implement protocol changes. Any recommendations were evaluated by the principal investigator and, when applicable, submitted for review and approval by the Research Ethics Committee.

### Frequency and plans for auditing trial conduct {29}

No formal independent audit was planned for this study, given its academic, single-centre design and minimal risk profile. Trial conduct was monitored through continuous internal oversight by the study team, with the aim of ensuring protocol adherence, data integrity, and participant safety.

Internal monitoring included periodic verification of study procedures, compliance with the schedule of interventions and assessments, and appropriate documentation of clinical data. Participant safety was additionally monitored by an independent physician acting as an independent safety monitor, as described in item {28a}, through scheduled monthly reviews of the collected data and reactive assessments in response to adverse events.

It was established that any relevant intercurrences, serious adverse events, or the need for substantial protocol modifications would be promptly reported to the Research Ethics Committee in accordance with applicable regulatory requirements. In the absence of serious adverse events or relevant protocol deviations, no additional periodic formal reports to the ethics committee were planned.

### Protocol amendments {31}

Any substantial protocol amendments, including methodological modifications, changes to outcomes, sample size, inclusion or exclusion criteria, or ethical aspects of the study, would be proposed by the principal investigator in an administrative and regulatory capacity and submitted in advance for approval by the Research Ethics Committee. No amendments would be implemented without prior ethical approval.

Approved amendments would be communicated and recorded in the applicable regulatory systems, including Plataforma Brasil and the Brazilian Registry of Clinical Trials (ReBEC), in accordance with prevailing regulatory requirements. When applicable, other relevant parties would be informed depending on the nature of the modification.

Any recommendations for protocol adjustments issued by the independent safety monitor for safety reasons would be communicated to the principal investigator without disclosure of outcome data or group allocation information. In such cases, the role of the principal investigator would be limited to conducting the necessary ethical and regulatory procedures for potential implementation, following approval by the Research Ethics Committee.

No substantial protocol amendments were required during the conduct of the study.

### Dissemination policy {8}

The results of this clinical trial will be disseminated through publications in peer-reviewed scientific journals and presentations at relevant scientific and medical conferences, aiming to communicate findings to healthcare professionals and the scientific community.

Key study results will also be reported in applicable public clinical trial registries, including the Brazilian Registry of Clinical Trials (ReBEC), in accordance with prevailing regulatory requirements.

A summary of the study results in plain language will be made available to participants upon request after completion of the study and final data analysis. When appropriate, relevant information may also be disseminated to the general public through institutional channels.

There will be no restrictions on the publication or dissemination of the study results, regardless of the nature of the findings, ensuring transparency and scientific integrity.

## Discussion

The conduct of this clinical trial entails limitations inherent to a prospective design with prolonged follow-up, particularly regarding participant retention at the 6- and 12-month assessments. In studies evaluating non-pharmacological interventions for chronic conditions such as genitourinary syndrome of menopause, maintaining follow-up after completion of treatment sessions represents a relevant challenge, as the perception of treatment completion may reduce participants’ motivation to attend subsequent clinical evaluations.

Although active follow-up strategies were planned and implemented, including periodic contacts and reinforcement of the importance of clinical monitoring for participant safety and data quality, losses during later follow-up cannot be entirely excluded. Attrition occurring at later time points may primarily affect the interpretation of outcomes related to the durability of the therapeutic effect and should therefore be interpreted with caution.

Additionally, considering the observed loss to follow-up, the final number of participants with primary outcome assessment may have been lower than initially planned, which may reduce the statistical power to demonstrate non-inferiority. Therefore, any conclusions should be interpreted with caution, particularly if non-inferiority is not established unequivocally.

To mitigate potential bias arising from loss to follow-up, the statistical analysis was planned according to the intention-to-treat principle, complemented by sensitivity analyses designed to assess the robustness of the findings under plausible attrition scenarios. Nevertheless, it is acknowledged that differential losses between groups or losses concentrated at later follow-up stages may influence the precision of long-term effect estimates, particularly in a study with a moderate sample size.

Another relevant methodological limitation is the absence of a control group treated with topical vaginal oestrogen, which is considered the standard therapy for genitourinary syndrome of menopause. The decision to adopt a comparative design between two non-hormonal therapies was based on ethical, clinical, and practical considerations, as a substantial proportion of women do not use or discontinue local oestrogen due to contraindications, safety concerns, personal preferences, or poor adherence. Accordingly, the present study was designed to address a specific and clinically relevant question: whether fractional microablative radiofrequency is non-inferior in clinical efficacy to fractional CO₂ laser, a more extensively studied technology, within the context of available non-hormonal treatment options.

Limitations related to blinding should also be considered in trials involving device-based therapies. Although the study was designed as double-blind, complete masking of participants cannot be fully guaranteed in non-pharmacological interventions, as procedural sensations or expectations associated with device-based treatments may influence subjective perceptions, including anxiety or expectancy effects. This challenge is inherent to procedural studies and is widely recognised in this field of research.

To minimise the potential impact of expectancy-related or anxiety-related effects, mitigation strategies were implemented, including outcome assessments performed by trained evaluators blinded to group allocation, the use of standardised clinical instruments with predefined scoring criteria, such as the VHIS, and strict standardisation of treatment protocols and participant information. These measures were intended to reduce measurement bias and minimise subjective variability associated with the interventions.

Although residual psychological influences cannot be entirely excluded, this limitation is common to device-based procedural trials and is unlikely to fully account for the observed clinical effects. The consistency of clinical outcomes and the similarity of responses between groups support the internal validity of the study despite these inherent challenges.

Taken together, the limitations discussed herein reflect methodological and operational challenges commonly encountered in non-pharmacological clinical trials and do not invalidate the relevance of the expected findings. Rather, they underscore the importance of interpreting the results in light of the study design and clinical context in which these interventions are applied, contributing to a transparent, balanced, and clinically meaningful assessment of the applicability of the data in routine practice.

## Trial status

Ethical approval for the study was granted on 19 April 2022. The screening phase of potential participants took place between 10 October 2022 and 13 February 2023, during which women underwent clinical assessment, completed complementary examinations, received the informed consent form, and returned with test results for eligibility verification. Formal inclusion of participants in the study (recruitment/enrolment) began on 15 February 2023. The interventions were conducted between March 2023 and September 2023, and participant follow-up was completed in September 2024.

The trial was retrospectively registered. At the time of submission of this protocol, the outcome database and all statistical analyses remained locked and inaccessible to the principal investigator and the research team. No outcome data were analysed prior to trial registration or prior to submission of the protocol to the journal.

During the intervention phase (day 1, day 30, and day 60), no missed visits or withdrawals were recorded.

At the primary outcome assessment (day 90), one participant permanently withdrew from follow-up, and seven participants missed the scheduled assessment, resulting in a total of eight missed evaluations at this time point. Missed visits at day 90 were attributed to personal reasons, family-related circumstances, or inability to attend within the predefined assessment window of approximately 30–40 days.

At the 6-month follow-up (day 180), a second permanent withdrawal occurred, in addition to two missed visits. When accounting for previous withdrawals, a total of four participants were without assessment at this time point. Permanent withdrawals occurred due to relocation to another city (one participant) and a voluntary decision to discontinue follow-up after achieving satisfactory clinical improvement (one participant).

At the 12-month follow-up (day 365), no additional missed visits were recorded beyond the two permanent withdrawals previously described.

These data relate exclusively to operational follow-up and participant attendance rates and did not require access to clinical outcome data or statistical analyses, which remained locked until after manuscript submission.

## Data Availability

Deidentified individual participant data, as well as the data dictionary and the statistical codes used in this study, are not publicly available due to ethical and confidentiality considerations. There is no plan to share individual participant data. Aggregated information and consolidated results may be made available upon reasonable request to the corresponding author, subject to approval by the Research Ethics Committee and compliance with ethical standards and participant confidentiality protections. Access to the final dataset is restricted to the principal investigator and to the relevant institutional bodies, in accordance with ethical requirements. There are no contractual agreements that limit investigators’ access to the data beyond these ethical requirements.

## Abbreviations

CO₂ laser: Carbon dioxide laser
FSFI: Female Sexual Function Index
GSM: Genitourinary syndrome of menopause
ICIQ-SF: International Consultation on Incontinence Questionnaire – Short Form
HE: Haematoxylin–eosin
ICF: Informed consent form
MAFRF: Microablative fractional radiofrequency
ReBEC: Brazilian Registry of Clinical Trials
RF: Radiofrequency
VAS: Visual analogue scale
VHIS: Vaginal Health Index Score

## References

[1] Portman DJ, Gass ML. Genitourinary syndrome of menopause: new terminology for vulvovaginal atrophy from the International Society for the Study of Women’s Sexual Health and the North American Menopause Society. Menopause. 2014;21(10):1063–8. 10.1097/GME.0000000000000329. 25160739

[2] Krychman M, Graham S, Bernick B, Mirkin S, Kingsberg SA. The Women’s EMPOWER survey: women’s knowledge and awareness of treatment options for vulvar and vaginal atrophy remains inadequate. J Sex Med. 2017;14(3):425–33. 10.1016/j.jsxm.2017.01.011. 28202319

[3] Palacios S, Nappi RE, Bruyniks N, Particco M, Panay N. The European Vulvovaginal Epidemiological Survey (EVES): prevalence, symptoms and impact of vulvovaginal atrophy of menopause. Climacteric. 2018;21(3):286–91. 10.1080/13697137.2018.1446930. 29553288

[4] Salvatore S, Nappi RE, Zerbinati N, et al. A 12-week treatment with fractional CO2laser for vulvovaginal atrophy: a pilot study. Climacteric. 2014;17(4):363–9. 10.3109/13697137.2014.899347. 24605832

[5] Perino A, Cucinella G, Gugliotta G, et al. Is vaginal fractional CO₂ laser treatment effective in improving vaginal atrophy symptoms in postmenopausal patients? Maturitas. 2015;80(3):296–301. 10.1016/j.maturitas.2014.12.006 25596815

[6] Gambacciani M, Palacios S. Laser therapy for the restoration of vaginal function. Maturitas. 2017;99:10–5. 10.1016/j.maturitas.2017.01.012. 28364861

[7] Kamilos MF, Borrelli GM. Radiofrequency in the treatment of genitourinary syndrome of menopause: a pilot study. Einstein (Sao Paulo). 2017;15(2):190–5. 10.1590/S1679-45082017AO4051. PMC5875158 29364367

[8] Slongo H, Henriques DCP, Ongaratto APN, et al. Microablative and non-ablative laser and radiofrequency treatment of genitourinary syndrome of menopause: a randomised controlled trial with four different energies. Maturitas. 2025. 10.1016/j.maturitas.2025.108737. 41061454

[9] Seganfredo IB, Bianchi C, Tacla M, et al. Comparison of promestriene with vaginal fractional CO₂ laser and radiofrequency treatments of genitourinary syndrome of menopause. Maturitas. 2024. 10.1016/j.maturitas.2024.108008. 38714422

[10] Cantarelli GC, Bianchi-Ferraro AMHM, Dedonatto C, et al. Clinical and histomorphometric evaluation of the vagina following treatment with CO2 laser, radiofrequency, and promestriene for genitourinary syndrome of menopause in breast cancer survivors on adjuvant therapy. Maturitas. 2024. 10.1016/j.maturitas.2024.108155. 39566128

[11] Torre PA, Fialho SCAV, do Icc VG, et al. Efficacy and safety of fractional microablative radiofrequency versus topical estriol in treating genitourinary syndrome of menopause: a pilot study. Int J Gynaecol Obstet. 2025. 10.1002/ijgo.70563. PMC12988376 41065268

[12] Chan A-W, Boutron I, Hopewell S, Moher D, Schulz KF, et al. SPIRIT 2025 statement: updated guideline for protocols of randomised trials. BMJ. 2025;389:e081477. 10.1136/bmj-2024-081477. PMC12035670 40294953

[13] Flight L, Julious SA. Practical guide to sample size calculations: non-inferiority and equivalence trials. Pharm Stat. 2016;15(1):80–9. 10.1002/pst.1716. 26604186

[14] Jang Y, Leung CY, Huang H. Comparison of symptom severity of genitourinary syndrome of menopause after carbon dioxide laser therapy versus vaginal estrogen therapy: a systematic review and meta-analysis. JAMA Netw Open. 2022;5(9):e2232563. 10.1001/jamanetworkopen.2022.32563. PMC9494191 36129710

